# Sexual Desire in Women: Paradoxical and Nonlinear Associations with Anxiety and Depressed Mood

**DOI:** 10.1007/s10508-022-02400-w

**Published:** 2022-09-19

**Authors:** Celeste Bittoni, Jeff Kiesner

**Affiliations:** Department of Development and Social Psychology, University of Padova, via Venezia 8, 35131 Padua, Italy

**Keywords:** Sexual desire, Anxiety, Depression, Women

## Abstract

The aim of the present study was to expand previous findings regarding paradoxical effects of negative mood on sexual desire. This was done by considering the full range of depressed mood and anxiety symptoms and using methods that are unaffected by recall bias and that don’t require participants to infer causal associations between their mood and sexual desire. A convenience sample of 213 university students completed daily questionnaires for approximately two months. Multilevel random-effects models were used to estimate average effects for the entire sample and to test for variability across participants in the associations between negative mood and sexual desire, controlling also for potential influences of the menstrual cycle. Previous findings showing that some women report decreased sexual desire and others increased sexual desire when depressed or anxious were confirmed. More importantly, for both depressed mood and anxiety, results demonstrated the presence of within-person paradoxical associations, whereby there were some women for whom both low and high levels of negative mood were associated with the same change (an increase or a decrease) in sexual desire. Related to these diverse response patterns, paradoxical associations between negative mood and sexual desire were also present at low levels of negative mood. The discussion underlines the importance of considering individual variability and multifactorial nonlinear models when studying sexual desire.

## Introduction

While there seems to be reasonable agreement that sexual desire is part of what guides us toward or away from sexual stimuli or activities (Levine, 2002), there is less agreement about what factors are most important in determining one’s changes in sexual desire. One factor that has caught the attention of sexuality researchers is negative mood, such as depression and anxiety. A puzzling aspect of these variables is that they have been associated with both positive and negative changes in sexual desire, in both men and women (Bancroft et al., 2003a, 2003b; Janssen et al., 2013). We believe this is especially relevant for women, who experience higher levels of mood symptoms and disorders (Altemus, 2006; Nolen-Hoeksema, 1987), whose mood and sexual desire may fluctuate across their menstrual cycle (Caruso et al., 2014; Kiesner, 2011;), and who report sexual desire-related issues more commonly than men (McCabe et al., 2016). Therefore, the goal of the present study is to examine the complex relations between sexual desire and depression and anxiety, using a sample of women.

It should be noted that, while there currently remains confusion and disagreement around terms like sexual interest, sexual motivation, sex drive, subjective sexual arousal, and sexual desire, all of which could be potentially interpreted as conveying subtle shades of a similar meaning, for both simplicity and consistency, we will use the term sexual desire to capture the general concept.

### Factors Influencing Sexual Desire—With a Focus on Negative Mood

Sexual desire and general sexual functioning have been found to be associated with many variables. For example, a developmental history of sexual abuse (Rellini & Meston, 2007; for reviews, see Brotto et al., 2010; Leonard et al., 2002) and the length of one’s current sexual relationship (Carvalheira et al., 2010; Klusmann, 2002) have been consistently associated with women’s sexual desire. Considering women’s sexual functioning more generally (i.e., not specific to sexual desire), evidence suggests that general emotional well-being (Bancroft et al., 2003a, 2003b, 2003c, 2003d), feeling desired and accepted by one’s partner (Graham et al., 2004), body-image satisfaction (Graham et al., 2004), sexual conservativism and cognitive interference (Nobre, 2009), psychiatric history (Brotto et al., 2010), and menopausal status (Cawood & Bancroft, 1996) are all important contributors. The influence of the menstrual cycle on sexual desire has also been theorized and widely tested (Bullivant et al., 2004; Roney & Simmons, 2013), with recent research suggesting that this association is very individual and that a general conclusion regarding the average woman is not justified (Kiesner et al., 2022).

Especially interesting are studies reporting a positive association between negative mood and sexual behavior and desire (Bancroft et al., 2003a, 2003b, 2003c, 2003d; Graham et al., 2004; Janssen et al., 2013; Lykins et al., 2006), which Bancroft et al. described as *paradoxical*. Although 35 years ago, Barlow (1986) had already proposed a model indicating that anxiety could have both positive and negative effects on sexual functioning, Barlow’s theory focused on sexually functional vs dysfunctional men and was based on research that pertained primarily to laboratory manipulations of threat and anxiety (see Meston & Gorzalka, 1995, and Palace & Gorzalka, 1990, for similar laboratory-based research on women). Thus, the relevance of anxiety to sexual desire in the general population and in relation to real-life experiences of anxiety remained largely unaddressed.

Regarding depression, although past research has suggested that people with major depression report decreased sexual desire, caused either by depression itself or by side effects of antidepressant medications (Lane, 1997; Montejo-González et al., 1997), the commonly held belief that depression exerts a negative influence on sexual desire has also been questioned. For example, Black et al., (1997) reported that a high percentage (31%) of both men and women with compulsive sexual behavior also showed a 6-month comorbidity for “major depression or dysthymia,” and Mathew and Weinman (1982) found that a sample of drug-free depressed patients demonstrated higher rates of both “loss of libido” and “excessive libido,” as compared to a non-depressed control group (although selection bias may have resulted in significant underestimates of sexual dysfunction among the control group). Overall, these data suggest that higher levels of sexuality are sometimes co-occurrent with mood disorders.

Qualitative research has also provided evidence for a paradoxical association between sexual desire and mood problems. Specifically, to identify factors that influence sexual desire and arousal, Graham et al., (2004) used focus groups in which women were asked to discuss what facilitates or interferes with the female sexual response, based on their direct or indirect experience. Among several factors discussed by participants as having either positive or negative effects on sexual arousal, such as self-image, or feeling desired vs feeling used, anxiety was found to have a paradoxical association with sexual arousal, with some women experiencing a heightened sexual response when anxious, while others reported the opposite. Although Graham’s study was primarily focused on female sexual arousal, many of the women in their study reported not clearly differentiating between desire and arousal; thus, it could be hypothesized that at least some of the factors indicated as influencing sexual arousal could also influence sexual desire. In fact, in the results presented, depression and anxiety were explicitly discussed as influencing both sexual arousal and sexual interest/desire.

More specific tests of the paradoxical effect of mood on sexual desire were conducted on gay and heterosexual men in a series of studies at The Kinsey Institute, using a measure that provides a more direct test of the association between negative mood and sexual desire. Specifically, the Mood and Sexuality Questionnaire (MSQ) asks participants to attribute changes in sexual desire and sexual performance to changes in anxiety and depression by asking “When you have felt depressed, what typically happens to [a] your sexual interest and [b] your sexual arousal?” and “When you have felt anxious/stressed, what typically happens to [a] your sexual interest and [b] your sexual arousal?”. Thus, participants are asked to make causal attributions regarding a possible association between mood and sexuality. Across studies, results showed that while most individuals report either no change or a decrease in sexual desire when experiencing negative mood, there are some individuals for whom sexual desire increases with higher levels of anxiety/stress and/or depression (Bancroft et al., 2003a, 2003b, 2003c, 2003d). In addition, for both gay and heterosexual men, those who reported experiencing increased sexual interest during negative mood states also reported a higher number of casual partners (Bancroft et al., 2003a, 2004), such that some men seem to be prompted to search specifically for casual sex when experiencing negative feelings. Finally, the positive association between sexual desire and negative mood seems to be especially present among sex addicts (Bancroft & Vukadinovic, 2004).

Most relevant to the current paper are results discussed in Lykins et al. (2006), who found that while 50.5% of women participants reported experiencing a *decrease* in sexual desire when depressed, 9.5% reported experiencing an *increase* in sexual desire. This paradoxical effect was even stronger for anxiety, given that 34% of women participants reported a decrease, while 23% reported an increase in sexual desire when feeling highly anxious. Nevertheless, the paradoxical effect of anxiety and depression on sexual functioning appears to be stronger in men than in women (Janssen et al., 2013).

Overall, past literature converges on the idea that both depression and anxiety show paradoxical effects on sexual desire—at high levels of negative mood. However, the studies discussed above have limitations that dictate caution when interpreting their results. For example, the Graham et al.’s (2004) study was based on qualitative methods in the context of focus groups, which could be biased by both the focus group members and facilitators. Also, although Bancroft et al., (2003a, 2003b, 2003c, 2004), Janssen et al. (2013) and Lykins et al. (2006) provide the most direct test of these effects, they also share an important limitation. Specifically, these studies asked participants to make causal attributions about a putative association between past mood states and past sexual desire, thus adding two layers of potential bias and measurement error. First, recall bias and poor reliability of retrospective self-reports have been noted for affective symptoms and non-sexual behaviors (Boschloo et al., 2013; Coughlin, 1990; Herrera et al., 2017), as well as specifically for sexual behaviors (Jaccard & Wan, 1995; Jaccard et al., 2002, 2004). Thus, results should be taken with caution until further tests can be conducted with more direct measures of changes in mood and sexual desire. Second, decades of research have shown that people’s causal attributions are frequently biased and unreliable (Mezulis et al., 2004; Miller & Ross, 1975; Sedikides et al., 1998), and although we could find no research specific to errors in causal attributions regarding sexual desire or behavior, they seem likely to exist, and therefore, caution should again be applied until these results are replicated without requiring individuals to make such attributions. Finally, a third limitation is that these studies have not tested what associations exist at low levels of negative mood. Although this issue might appear theoretically predictable and less practically relevant, evaluating the effect across the full range of the same emotion would guide us toward a better general understanding of the relationships between sexual desire, anxiety, and depression.

### Current Study

The aim of the present study was to test and extend the hypotheses and findings of Bancroft et al., (2003a, 2003b, 2004), Lykins et al. (2006), and Janssen et al., (2013), with a sample of women. The exclusive focus on women is because the data used come from a study on menstrual cycle-related symptoms and because low sexual desire is a common problem among women (Parish & Hahn, 2016; Worsley et al., 2017). We address the main limitations described above, namely the use of retrospective reports and requiring participants to make causal attributions regarding the association between negative mood symptoms and sexual desire. Moreover, because past studies have focused on testing for paradoxical effects only at high levels of negative mood, we will examine these associations across the full range of these mood symptoms.

The above limitations are addressed in three ways. First, day-to-day reports, rather than retrospective recall, are used to measure daily changes in mood and sexual desire. Second, to avoid asking participants to make causal attributions regarding negative mood and sexual desire, we used separate items and repeated measurements, without asking participant to infer any type of causation on their own. Third, we tested for associations between mood and sexual desire across the full range of negative mood symptoms. In addition to these methodological improvements, the present analyses are based on a large sample (*n* = 213) of women who participated in a study on the menstrual cycle in which they were asked to provide daily reports for two full menstrual cycles (approximately 2 months). Thus, with this high frequency of measurement over an extended time period and a large sample, we were able to model individual change across time in all measured variables, as well as covariation in the observed changes. These methodological advancements present significant improvements, and we believe will provide robust tests of the research questions.

Finally, given that fluctuations in depression and anxiety (Kiesner, 2011; Kiesner et al., 2016), as well as sexual desire (Kiesner et. al., 2022), can be associated with the menstrual cycle for some women, it is possible that observed associations between mood and sexual desire could be confounded with menstrual cycle effects. Therefore, to rule out the possibility that any observed associations between mood and sexual desire are the result of the menstrual cycle influencing both types of variables (i.e., third variable confound), we statistically controlled for cyclical effects of the menstrual cycle on changes in sexual desire.

## Method

### Participants

The data presented in this paper are from a larger study focusing on various symptoms of the menstrual cycle and from which previous reports have been published (data collected from 2007 to 2008). These earlier reports have focused on menstrual cycle-related changes in mood symptoms (Kiesner, 2011), headaches (Kiesner & Martin, 2013), and sleep (Van Reen & Kiesner, 2016). However, only the current study, and a study in preparation (Kiesner et al., 2022), have examined sexual desire, and these two studies address very different questions (e.g., Kiesner et al., 2022 presents a detailed analysis of menstrual cycle effects on sexual desire, including how physical and psychological symptoms of the menstrual cycle may influence sexual desire). So, although there is significant overlap in the methods section of the present report and those of earlier reports, the research questions addressed are very different.

Participants were 213 female university students with a mean age of *M* = 21.29 years (*SD* = 4.01), of which *n* = 209 (98%) were Italian (see Table 1 for other demographic information). All first-year female psychology students were asked to participate, and efforts were made to include women both with and without menstrual difficulties. The main inclusionary criterion were that participants have a natural and regular menstrual cycle. Thus, individuals could not participate if they were using hormonal contraceptives or therapy. Preexisting illness (physical or psychological) was not used as an exclusionary criterion, and thus, those who had been diagnosed with a psychological or medical condition for which they had been, or were being treated, were welcomed to participate. However, participants with a seasonal illness (cold/flu) at the time of their next menstrual flow were asked to wait until it had passed before starting (e.g., waiting till the onset of a subsequent menstruation). Participation was anonymous, voluntary, and did not result in compensation. The Ethics Committee of Psychological Research, of the University of Padova, approved this study, and all participants signed an informed consent.

**Table 1:** Descriptive and demographic characteristics

Variable	Mean (S.D.) n (%)
Age	21.29 (4.01)
Age at menarche	12.30 (1.45)
Had children	2 (1%)
Smoker	48 (23%)
Reported illness	18 (8.5%)
Illness Name (n with named illness; treatment)	Allergy (1; antihistamine) Anorexia (1) Anxiety (2; SSRI, benzodiapine) Asma (1; cortisone, antihistamine) Cardiac (1) Depression (1) Epilepsy (2; depakene, clonazepam, lamotrigine, levetiracetam) Herniated Disk (1) HPV (1) Hypertension (1; enalapril) Migraines (1; Difmetré: indometacin, prochlorperazine maleate) PCOS (1) Stargardt (1) Tachycardia (1) Thyroid (1; levothyroxine) Tooth Extraction (1; antibiotics)

Recruitment was conducted at the end of lectures in first-year psychology classes, after all male students were asked to leave the lecture hall. A brief explanation of the study was given, without providing specific information regarding study hypotheses. A central point that was emphasized during the explanation was the importance of including women who have very different experiences during the menstrual cycle and that it would be equally important for women with and without menstrual difficulties to participate. For example, it was specifically stated that for the success of the study it was equally as important to have women who experience menstrual cycle-related changes (physical or psychological) and those who experience no changes whatsoever. This was emphasized to reduce self-selection bias that could lead to more individuals with significant cyclical changes choosing to participate. Other points that were emphasized during the explanation were: (1) the personal nature of the questions and (2) the degree of participation required (daily questionnaires for two menstrual cycles). These points were emphasized to avoid surprise on the part of participants. The overall presentation, including questions and responses, lasted approximately 15 min.

Of the 897 individuals who were asked to participate, 320 (36%) responded positively and 577 (64%) responded negatively. Those who responded negatively were given the possibility to anonymously indicate why they chose to not participate, using a single-question multiple-choice format response. The distribution of responses was as follows: 20% did not have a regular menstrual cycle; 40% were using oral contraceptives or some other hormonal-based treatment; 22% had no computer access; 5% were not interested; 6% some other reason; and 7% gave multiple reasons. Of the 320 individuals who agreed to participate, 213 (67%) participated for the full study, providing data for two cycles. The data from these 213 participants are analyzed in the present study.

Research assistants met each participant individually to provide an explanation and demonstration of the online data collection procedure and to review all questions and provide explanations when needed.

The average length of the two menstrual cycles was *M* = 29.57 days for cycle 1 and *M* = 30.48 days for cycle 2 (average length of two consecutive cycles *M* = 60.05 days). The average number of questionnaires for each participant was *M* = 55.09. Thus, on average, participants missed only 5 of the daily questionnaires across the two menstrual cycles, and a total of 11,735 questionnaires were included in the following analyses.

### Measures

#### Online Questionnaire and Procedure

With the use of an individual password, participants had access to an online questionnaire. All questions referred to the last 24 h. Participants were asked to begin completing questionnaires on the first or second day of menstruation and to indicate on which day they were starting. Because data collection was conducted using an online questionnaire, the time and date of completion were automatically recorded and saved with each questionnaire. Participants were asked to complete one questionnaire each day. However, if they were not able to do so, or accidentally missed a day, the online questionnaire also allowed participants to complete one questionnaire for the prior day, and one questionnaire for the actual day. To control for this, the first question on each questionnaire was whether it was in relation to “yesterday” or “today.” Participants were asked not to go back more than one day (“yesterday”). Of the total number of questionnaires included in the present analyses (*n* = 11,735), 74% were completed on the actual day, and 26% were completed for “yesterday.”

#### Sexual Desire

Sexual desire was measured with two questions regarding increased in sexual desire and decreased sexual desire. Both questions were preceded with “In the last 24 h:”, then the questions were “…did you experience an increase sexual desire?” and “…did you experience a decrease in sexual desire?”. Responses were given on a 5-point response scale ranging from 1 = “*Not at all*” to 5 = “*Very much.*” In the present paper, we use a difference score (increased sexual desire–decreased sexual desire) to best capture a similar bipolar measure of sexual desire as used by Bancroft et al., (2003a, 2003b, 2003c, 2004), Janssen et al. (2013), and Lykins et al. (2006). The final score had a range of − 4 to + 4 (*M* = 0.54, *SD* = 1.41), with negative scores indicating a decrease in desire and positive scores indicating an increase in desire.

#### Depressed Mood

The measure of depressed mood was based on the following 4 questions (preceded with “In the last 24 h…:”): “…did you feel down?”; “…did you feel depressed?”; “…did you feel sad?”; “…did you have crying spells?”. Responses were given on a 5-point response scale ranging from 1 = “*Not at all*” to 5 = “*Very much.*” The depressed mood score was the mean of those four non-standardized items. This was done separately for each day.

#### Anxiety

The measure of anxiety was based on the following 2 questions (preceded with “In the last 24 h…:”): “…did you feel anxious?”; “…did you feel tense or nervous?”. Responses were given on a 5-point response scale ranging from 1 = “*Not at all*” to 5 = “*Very much.*” The anxiety score was the mean of those two non-standardized items. This was done separately for each day.

#### Menstrual Cycle

Because data were originally collected to investigate changes related to the menstrual cycle, the time and date of completion for each questionnaire were recoded to represent the proportion of each cycle that had passed since the first day of that cycle (day within cycle/total number of days in that cycle). Therefore, all participants, regardless of how many days their cycle lasted, were put on the same metric, ranging from 0 to 1 for each cycle (a 1 was then added to all days in the second cycle). The time variable ranged from 0 to 2, with 0 corresponding to the first day of the first cycle, 1 corresponding to the last day of the first cycle, and 2 corresponding to the last day of the second cycle. As already stated in the introduction, even though the present study is not focused on changes in sexual desire across the menstrual cycle, we control for the potential effects of the menstrual cycle to avoid a third variable confound. This was done by including the cosine function of time (i.e., the menstrual cycle) in all analyses, which allowed us to subtract the cyclical changes in sexual desire attributable to the effect of the menstrual cycle.

### Data Analysis

The aims of the following analyses were to (1) estimate the average effects (across the entire sample) of depressed mood and anxiety, on sexual desire; (2) test for individual differences across women in those same effects; and (3) identify diverse patterns, across women, in those effects. This was made possible because we had daily measures of anxiety, depressed mood, and sexual desire for approximately two months for each participant, and thus, we were able to investigate how variations in mood are associated with variations in sexual desire for the entire sample together (average effects), as well as for each individual participant separately (individual differences). With individual-level estimates of these associations, we were also able to use cluster analyses to group women based on their patterns of associations between sexual desire and the two mood symptoms. The technical aspects of these analyses are presented below.

Moreover, because we had daily reports across two full menstrual cycles, and because the menstrual cycle can influence both mood and sexual desire, it was important to statistically control for the effect of the menstrual cycle on sexual desire. This was done by regressing sexual desire on the cosine function of time (across two menstrual cycles), thus capturing menstrual cycle-related changes in sexual desire and removing this possible confound from the main analyses. However, because the focus of the present study is not on the menstrual cycle, we will not provide further explanation of these analyses (besides the results in Table 2). Rather, the reader is referred to Kiesner, et al. (2016) or Kiesner et al. (2022), for a detailed explanation of cosine regressions for studying the menstrual cycle (Kiesner et al., 2016), and how they apply specifically to sexual desire (Kiesner, et al., 2022).

**Table 2:** Coefficients and test statistics for multilevel model predicting sexual desire difference scores (Increase–Decrease) with fixed effects random intercepts, and random slopes

Predictor	Coefficient	Variance component	*t*	95% CI Wald *p*
Depressed mood				
*Fixed effects*				
Cosine	−.22		− 7.06***	
Depressed mood	−.40		− 7.78***	
Depressed mood ^2^	.08		2.31*	
*Random effects*				
Intercept		.43		.35−.52***
Cosine		.16		.12−.20***
Depressed mood		.27		.16−.37***
Depressed mood ^2^		.09		.05−.13***
Residual		1.37		1.34−1.41

Anxiety				
*Fixed effects*				
Cosine	−.23		− 7.30***	
Anxiety	−.18		− 6.54***	
Anxiety ^2^	.04		1.80	
*Random effects*				
Intercept		.41		.33−.50***
Cosine		.16		.12−.21***
Anxiety		.07		.04−.09***
Anxiety ^2^		.02		.01−.03**
Residual		1.41		1.37−1.45

* *p* < .05; **p < .001; ***p < .0001

Fixed Effects *df*s for Depressed Mood: Cosine *df* = 201.7; Depressed Mood *df* = 150.4; Depressed Mood^2^ *df* = 127.8

Fixed Effects *df*s for Anxiety: Cosine *df* = 199.9; Anxiety *df* = 172.4; Anxiety^2^ *df* = 156

Data were analyzed in two steps. First, multilevel (mixed) models were conducted using JMP Pro 15 software (SAS Institute, 2019). In these analyses, we consider the following three effects: cyclical effects of the menstrual cycle (cosine of time), linear effect of mood (depressed mood and anxiety), and quadratic effect of mood (depressed mood and anxiety). All three of these effects were treated both as fixed effects (e.g., average effects across the full sample) and as random effects (e.g., with the effect estimated separately for each individual participant, and testing for individual differences across participants). Because participants had different mean levels and different minimum and maximum values of mood symptoms, each mood variable was mean-centered within participant prior to creating the quadratic effect. This eliminates confounding of within-person and between-person effects and reduces multicollinearity between the linear and quadratic terms. This also means that the effects of mood are interpreted as relative to the individual’s average level, rather than regarding absolute values of mood on the original scale. Note that, sexual desire scores also show mean-level differences across participants, but those differences are specifically modeled in the analyses, so centering that variable is neither useful nor desired.

#### Linear and Nonlinear Associations

Regarding the effects of depressed mood and anxiety, we include the linear and quadratic (curvilinear) effects of both, testing whether depressed mood and anxiety are associated with changes in sexual desire, and if so, whether those associations are linear or nonlinear. Moreover, both the linear and quadratic effects are included as both fixed and random effects (fixed effects are the average effect for the entire sample, whereas random effects are a test of individual differences in that effect across participants). When the linear and quadratic effects of mood are included in the model simultaneously, they capture both linear and nonlinear associations that could have a variety of shapes, including linear slopes, exponentially accelerating or decelerating slopes, and - or   -shaped slopes, representing the association between mood symptoms and sexual desire. Based on past results presented by Bancroft et al., (2003a, 2003b, 2003c, 2004), Janssen et al. (2013), and Lykins et al. (2006), it was expected that for high levels of depressed mood and anxiety there would be some women who experience an increase in desire and other women who experience a decrease in desire. However, those previous studies only considered changes in desire at high levels of negative mood symptoms, not at lower levels of negative mood symptoms, whereas in the present study we are able to examine changes in desire across a range of mood symptoms within each individual.

#### Cluster Analysis

To group participants based on their individual associations between sexual desire and mood symptoms, we conducted cluster analyses based on individual regression slopes. To do so, we first conducted multiple regression analyses separately for each participant including the linear and quadratic (curvilinear) effects of either anxiety or depressed mood as predictors, and sexual desire as the dependent variable. The two standardized regression coefficients (β’s for the linear effect and quadratic effect) from these analyses were then saved separately for each individual. We next used these saved regression coefficients as variables in a two-step cluster analysis (repeated separately for depressed mood and anxiety). First, to determine the optimal number of clusters, the scree plot and dendrogram resulting from a hierarchical cluster analysis (Ward’s method) were examined. Second, a *K*-means cluster analysis was used to classify each individual into a cluster based on the shape of their association between mood and sexual desire (e.g., positive, negative, linear, nonlinear). Because past research on these associations has only considered the paradoxical effects of mood at high levels of mood distress, there is no prior research to guide hypothesis development for the overall shape of association between mood symptoms and sexual desire when a full range of mood change is considered. Therefore, no a priori hypotheses were made regarding the number of clusters, or the shape of association between mood and sexual desire.

It should be noted that conducting these cluster analyses is justified only if there are significant individual differences across participants in the associations between mood symptoms and sexual desire (i.e., random effects of the slopes). Also, the goal of these cluster analyses is only to provide descriptive and graphic summary of the observed individual differences in a way that can be used for interpretation and insight.

## Results

### Results—Summary

**Fig. 1:**
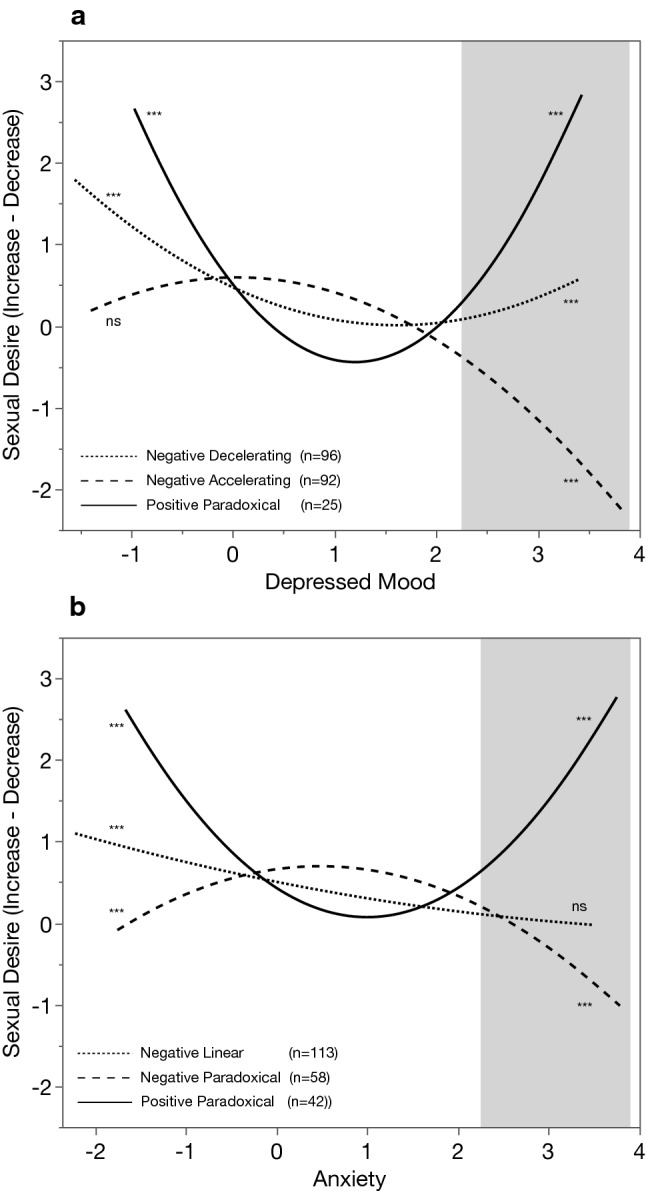
Curvilinear associations between negative mood scores (depressed mood and anxiety) and changes in sexual desire, plotted separately for clusters of women demonstrating diverse patterns of association between these variables. Asterisks and “ns” indicate significance as presented in Table 3; those on left are for linear effects (overall linear trend across the full range of mood symptoms), and those on the right are for the quadratic effects (curvature of the regression line)

To facilitate comprehension, in the current paragraph we give a summary using non-technical terms. Corresponding to the data analytic aims discussed above, there are three main results that will be discussed (note that, results regarding the menstrual cycle are included in the tables but will not be discussed in the text). First, there is a significant average effect across the entire sample of both depressed mood and anxiety on sexual desire, which is defined by an overall decrease in sexual desire as negative mood symptoms increase (see *fixed effects* in Table 2). Second, there is a high level of individual variability across women in these associations (e.g., women are different from each other), indicating that average effects across the entire sample do not adequately capture the association between sexual desire and mood (see *random effects* in Table 2). Third, for both anxiety and depressed mood, participants can be categorized into one of three clusters that show different patterns of associations between sexual desire and negative mood. These patterns of change are presented in Fig. 1 and can roughly be described in the following ways: one group of women who demonstrates a -shaped association between negative mood and changes in sexual desire, with decreases in sexual desire observed at both high and low levels of negative mood; and a third group of women that falls in between, with a more negative slope across the full range of negative mood (Fig. 1). These patterns of change, as well as all other results, are discussed in detail below.

### Results—Detailed

Results from the multilevel analyses are presented in Table 2. In the top half of Table 2 are the results for depressed mood, and in the bottom half of Table 2 are the results for anxiety. Within each set of analyses, the fixed effects are presented in the top half and the random effects in the bottom half. The fixed effects represent the average effects across the entire sample for the cyclical effect of the menstrual cycle (cosine), and the linear and curvilinear (quadratic) effects of each mood symptom. For the fixed effects of mood, the non-standardized regression coefficients are presented and can be interpreted in the following way: the linear effect represents the overall linear trend across the full range of mood symptoms, whereas the quadratic effect provides an index of curvature of the line across the range of mood scores. Note that, because these coefficients are not intuitively informative, readers are encouraged to review Fig. 1 to understand the shape of the actual regression slopes that are discussed below.

Random effects, instead, test for variability across individuals, and a significant random effect indicates that there is variability around the average/fixed effect for the sample. That is, significant random effects indicate that individual women are different from each other with regard to that effect, and thus, the average effect for the sample could not be considered an adequate description at the individual level. Note that, for the random effects, there are no regression coefficients, but rather variance components are used, which are an index of variance that can be attributed to individual differences across women in that specific effect.

As presented elsewhere (Kiesner et al., 2022), the cosine effect (menstrual cycle effect) was significant and negative, indicating that on average, women demonstrate a mid-cycle peak in sexual desire. Note, however, that the random effects of the intercept were significant, indicating that women are significantly different from one another in their average level of sexual desire, and the random effects of the menstrual cycle were also significant, suggesting differences between-women in how sexual desire changes across the menstrual cycle. These effects are addressed in detail in Kiesner et al., 2022 and will not be discussed further in the present paper.

Results for depressed mood (top half of Table 2) show that the fixed effects for both the linear and quadratic terms of depressed mood were significant, with a negative linear slope followed by a positive quadratic slope. Thus, on average, for the entire sample, there is an overall negative association between depressed mood and sexual desire, with a positive curvature in the slope as depressed mood increases. However, the tests of random effects showed that significant differences exist across individuals in both the linear and quadratic effects of depressed mood, and thus, the average effects cannot adequately describe these associations across individual participants. That is, individual women are different from each other in their relation between depressed mood and sexual desire, and one curve for the entire sample is not adequate to describe this relationship. This variability across women will be addressed below.

Results for anxiety (bottom half of Table 2) present a similar picture, with the exception that the quadratic fixed effect of anxiety was not significant. Thus, on average, for the entire sample, there is only a significant negative association between anxiety and sexual desire. However, both random effects of anxiety (linear and quadratic) were again significant, indicating that significant differences exist across individuals in both the linear and quadratic effects of anxiety, and thus, the average effects cannot adequately describe these associations across individual participants.

#### Cluster Analyses

Given the above set of analyses, cluster analyses were used to test whether women could be classified based on their individual pattern of association between mood symptoms and sexual desire. For these analyses, the standardized regression coefficients for the linear and quadratic effects were saved separately for each individual (taken from separate individual regression analyses) and used as variables in a two-step cluster analysis. For both mood symptoms, the hierarchical cluster analysis (non-standardized) indicated the existence of three groups. Next, using a *K*-means cluster analysis, each individual was classified into one of these three trajectory groups.

Regression lines showing each cluster’s association between levels of mood symptoms and changes in sexual desire are presented in Fig. 1, and regression analyses testing for linear and quadratic effects of each mood symptom, separately for each cluster group, are presented in Table 3. Considering the slopes (Fig. 1) and significance tests regarding each cluster (Table 2), descriptive labels were given to each of the cluster groups. When considering the association between depressed mood and sexual desire, the largest cluster was the *Negative Decelerating* cluster (*n* = 96; decelerating is used in the sense that the negative slope become less negative) that is characterized by a significant negative linear trend qualified by a significant positive change in the slope, indicating that the initial negative slope flattens out, and possibly becomes positive. The second largest cluster was the *Negative Accelerating* cluster (*n* = 92; accelerating is used in the sense that the negative slope become increasingly negative) that is characterized by an overall flat slope (nonsignificant) qualified by a rapidly accelerating negative slope. Finally, a *Positive Paradoxical* cluster (*n* = 25), which was named as such because within the same group there appears to be a paradoxical effect, whereby increases in desire are associated both with high and low levels of depressed mood.

**Table 3:** Linear and quadratic coefficients for pattern groups of depressed mood and anxiety

Depressed mood			
	Negative accelerating	Negative decelerating	Positive paradoxical
Depressed mood	.04	−.53***	−1.34***
Depressed mood^2^	−.21***	.16***	.59***

Anxiety			
	Negative linear	Negative paradoxical	Positive paradoxical
Anxiety	−.21***	.16***	−.56***
Anxiety^2^	.02	−.16***	.29***

*** *p* < .0001

In all models, the fixed effect of cosine, as well as the random effects of intercept and cosine, were also included but are not presented in the table. In all cases, those effects were significant and in the same direction as in the initial analyses presented in Table 2

When considering the association between anxiety and sexual desire, the largest cluster was the *Negative Linear* cluster (*n* = 113) that is characterized by a significant negative linear trend and a nonsignificant quadratic effect, indicating that the same basic trend applies across all levels of anxiety. The second largest cluster was the *Negative Paradoxical* cluster (*n* = 58) that is characterized by an overall positive slope qualified by a significant negative curvature in the slope, thus showing a paradoxical effect within this group, whereby low levels of sexual desire are associated with both low and high levels of anxiety. Finally, also for anxiety, a *Positive Paradoxical* cluster (*n* = 42) was observed, in which, within the same group, there is a paradoxical effect, whereby higher levels of desire are associated both with high and low levels of anxiety.

It should be noted that the replication with the past research (Bancroft et al., 2003a, 2003b, 2003c, 2004; Janssen et al., 2013; Lykins et al., 2006) is evident in the right side of Fig. 1a and b, when depressed mood and anxiety are high (shaded area of these figures). The novel findings regarding the broader associations between mood symptoms and sexual desire, on the other hand, are only evident when considering the full range of mood symptoms, and thus, the full curvilinear slopes are presented in Fig. 1a and b.

#### Missing Data

Because some participants completed fewer daily reports than expected (the length of their two menstrual cycles was greater than number of daily reports), we tested whether the number of missing reports within each participant was associated with level of depressed mood, anxiety, and sexual desire (correlations), and the clusters for depressed mood and anxiety (ANOVAs). None of these associations were significant (all *p*s > 0.42). Thus, the number of missing daily reports for each participant was unrelated to their level of depressed mood, anxiety, sexual desire, or cluster membership.

## Discussion

The present study expanded previous findings of paradoxical associations between sexual desire and both anxiety and depressed mood. Specifically, whereas previous studies examined paradoxical effects only at high levels of negative mood (Bancroft et al., 2003a, 2003b, 2003c, 2004; Janssen et al., 2013; Lykins et al., 2006), in the present study we examined how sexual desire changes across the full range of depressed mood and anxiety. Two important and novel findings emerged. First, a similar *between-groups* paradoxical association between mood and sexual desire was observed also at low levels of mood symptoms, for both depressed mood and anxiety (see left side of Fig. 1a and b). Thus, some women reported an increase, whereas others a decrease in their sexual desire, also at low levels of depressed mood and anxiety. This had not been previously tested. Second, some women showed the same change in sexual desire (increase or decrease) at both high and low levels of mood symptoms (see - and -shaped slopes in Fig. 1a and b). That is, women in the positive and negative paradoxical groups demonstrated a *within-person* paradoxical association between sexual desire and negative mood symptoms, in that they reported the same change in sexual desire for opposite levels of negative mood, e.g., an increase in sexual desire when both anxious and not anxious, with low desire at their midpoint of anxiety.

In addition to these novel findings, we were also able to replicate the between-groups paradoxical association between high levels of negative mood and sexual desire. These replicated paradoxical associations can be observed on the right side of Fig. 1a and b, highlighted in grey, where some women demonstrate a decrease in sexual desire and others an increase, when experiencing high levels of negative mood. These results are a clear replication of past work by Bancroft et al., (2003a, 2003b, 2003c, 2004), Janssen et al., (2013), and Lykins et al. (2006). Moreover, an important aspect of the replication of previous results is the similarity in effect sizes for anxiety, relative to depressed mood. Specifically, the Lykins et al.’s study found that, among women, the paradoxical association (i.e., an increase in desire at high levels of negative mood) is more common for anxiety (23%) than for depression (9.5%), whereas, in the present study, using a very different methodology, a similar difference was observed, with the paradoxical association again being more common for anxiety (19.7%) than for depression (11.7%). Thus, this difference between anxiety and depression in the observed paradoxical associations appears robust across very different methods and samples.

The remainder of this discussion is divided into two main sections. We start with the simplest findings that have been observed in previous studies, focusing on between-group paradoxical associations at the high end of negative mood. The second section will then address the two novel and inter-related findings of the present study: the within-person paradoxical associations, and in doing so, the paradoxical associations observed also at low levels of negative mood.

### Replication of Between-Groups Paradoxical Associations

Given previous research showing an association between mood disorders (depression and anxiety) and decreased sexual desire (Angst, 1998; Beck, 1967; Cassidy et al., 1957; Figueira et al., 2001; Schreiner-Engel et al., 1986), it was those who reported increased sexual desire when depressed or anxious who Bancroft et al., (2003a, 2003b, 2003c, 2004) considered to be “paradoxical.” Moreover, research showing that depression is associated with diminished interpersonal behavior (Youngren & Lewinsohn, 1980), social withdrawal (Girard et al., 2014), and anhedonia and fatigue (Gotlib et al., 1995), further suggests that increased sexual desire associated with depression is paradoxical. To understand what drives the paradoxical effect of negative mood on sexual desire, Bancroft et al. conducted qualitative interviews with heterosexual men (Bancroft et al., 2003b) and gay men (Bancroft et al., 2003c) to identify specific mechanisms for why people respond to negative affect with such different sexual responses. Their results suggested that emotional regulation strategies, such as seeking intimacy and self-validation, likely play important causal roles in creating this paradoxical association. To the extent that the qualitative data from men can be generalized to women, the same causal mechanisms may apply to our data.

It is also important, however, to contextualize depression in terms of its specific causes and in terms of diverse coping strategies that people may use when depressed. For example, consider two women who both score high on depression, but for different reasons. Woman A feels depressed because of chronic interpersonal conflict at work, for which she risks losing her job. Woman B, instead, is living in an unhappy romantic relationship from which she sees no way out. Sexual desire of woman A could be unaffected by negative mood if she is able to correctly attribute her depressed mood to the work context and limit its effects on her romantic relationship. On the contrary, because woman B’s depressed mood is strictly linked with her romantic partner, she is likely to actively avoid sex and report low levels of sexual desire. Across these two scenarios, high levels of self-reported depression will have very different associations with sexual desire.

Moreover, different coping strategies could be used for the situations that were described above. For example, woman A might blame her romantic partner for work difficulties (e.g., scapegoating), or vent her negative mood accumulated during the workday towards her romantic partner, thus creating dyadic conflict and indirectly lowering sexual desire. Similarly, women B could use sex as a coping strategy to improve the relationship with her partner, making it appear that her sexual desire is increasing rather than decreasing. Thus, although emotional regulation is likely an important coping strategy that was easily identifiable by participants in Bancroft et al.’s (2003b) qualitative study, and important for understanding the paradoxical association, there may be other coping strategies that are not directly focused on affect regulation.

In addition to interpreting their results in the context of affect regulation, Bancroft et al. (2003b) also discussed their findings in light of the dual control model of sexual response (Bancroft, 1999; Bancroft & Janssen, 2000). Results from heterosexual men support the idea that individual differences in sexual excitation and inhibition (the key components of the dual control model) are associated with the observed paradoxical effects (Bancroft et al., 2003b), but weaker evidence was found for gay men (Bancroft et al., 2003c) and women (Lykins et al., 2006). Thus, to help understand paradoxical effects of mood on sexual desire, the dual control model may need to be adapted when applied to individuals other than heterosexual men. More specifically, sexual inhibition in women cannot be conceptualized in the same way as for men (Graham et al., 2004, 2006), and stimuli that can ignite sexual desire differ between genders (Meston & Buss, 2007). For example, women cited “being used by the partner” or “fear of ruining their reputation” as inhibiting factors (Graham et al., 2004), and partner’s acceptance of their body as essential to excitation. On the other hand, fear of performance failure (Bancroft et al., 2009; Bancroft & Janssen, 2000; Janssen et al., 2002) is believed to be an important inhibiting factor in men, who mainly focus on physical appearance and desirability of a partner to augment sexual excitation (Meston & Buss, 2007). One approach to making the dual control model relevant to the paradoxical effects for individuals other than heterosexual men is to modify the measurement of the sexual inhibition/excitation constructs (SIS/SES) central to the Dual Control model. Lykins et al., (2006), in fact, did use a modified version of the SIS/SES, but considering results from Graham et al., (2004), simply changing the terminology, for example, to indicate genital arousal (i.e. vaginal lubrication rather than erection) might not be sufficient.

Regarding the paradoxical effect associated with high levels of anxiety*,* we can again consider Bancroft et al. (2003b), who suggested that the paradoxical effect with anxiety may be attributable to tension release and the calming effect of sexual pleasure in combination with “excitation transfer” (a concept introduced by Zillman, 2008) of general anxiety to sexual tension/arousal. According to Zillman (2008), the physiological changes occurring in response to anxiety take time to resolve and can therefore persist even after the cognitive response to anxiety has passed. Consequently, this “residual excitation” could influence the perception and interpretation of subsequent, non-anxiety provoking, stimuli. Relatedly, because there are associations between anxiety and sympathetic nervous system activity (Hoehn-Saric & McLeod, 2000), anxiety and provoked sexual arousal (Palace & Gorzalka, 1990), and sympathetic arousal and provoked sexual arousal (Meston & Gorzalka, 1995), it has been hypothesized that an increase in sympathetic activity due to anxiety facilitates sexual response (Bradford & Meston, 2006; Meston & Gorzalka, 1995; Palace & Gorzalka, 1990). In addition, we suggest that this occurs by lowering the threshold required to be receptive to sexual cues, and thus, perceiving an increase in sexual arousal. Although this provides an explanation for why some people may report increased sexual desire when feeling anxious, it does not help to explain the existence of individual differences: why some women respond in the expected or non-paradoxical way, and others respond in a paradoxical way.

We propose two possible explanations. One possibility regards the causes of anxiety. Similar to what was discussed for depression, different causes of increased anxiety could lead to different consequences for women’s sexual desire. For example, if a woman’s experience of anxiety is linked to problems with her current sexual partner, it is likely that thinking about sex would be associated with the cause of, rather than a solution to, anxiety. Therefore, she might want to avoid, rather than search for sex, feeling even more anxious. On the other hand, if a woman’s anxiety is related to stress for an upcoming exam session, her sexual desire might increase in response to the need of having a pleasurable space where she can release tension, e.g., a safe anchor behavior.

The second possibility regards the individual’s general level of sexual functioning. More than 30 years ago, in a review of the literature, Barlow (1986) noted that individuals with a healthy sexual functioning, as compared to those who struggle with sexual dysfunctions, respond in opposite ways to the same moderate-to-high level of anxiety. Considered in the context of the present study, it could be hypothesized that the opposite effects found for women in the positive paradoxical vs negative paradoxical groups are associated with general levels of sexual functioning, with women in the negative paradoxical group having a poorer general level of sexual functioning compared to women in the positive paradoxical group. Although this does not provide a specific causal mechanism, it does provide a potential correlated construct that may help us explain these paradoxical effects and that should be considered in future research.

### Within-Person Paradoxical Effects

In the following section, we address the two novel and interrelated findings that emerged in the present study: (1) the within-person paradoxical associations across the full range of negative mood symptoms, found for both depressed mood and anxiety, and (2) the paradoxical effects found also at low levels of negative mood. It should be noted that the existence of the original paradoxical effect, as well as the newly found paradoxical effect at the low end of negative mood, are both driven by the existence of groups of women who show the within-person paradoxical associations. Therefore, in the following discussion, we will focus on mechanisms underlying the within-person paradoxical associations. Thus, we will try to explain why some women present a similar increase (or decrease) in sexual desire for both low and high levels of negative mood.

One possibility is that the same changes in sexual desire, under very different mood states, are driven by different mechanisms at the different extremes of mood. For example, high levels of desire associated with low levels of depressed mood may best be conceptualized in terms of “celebratory” effects of positive mood/happiness. That is, it may be happiness, not low negative mood, that drives the increase in sexual desire when women report low levels of negative mood. For example, Kiesner et al. (2022) found that day-to-day changes in happiness were significantly associated with day-to-day changes in sexual desire, even after controlling for the concurrent effects of depressed mood. On the other hand, an increase in sexual desire associated with high levels of depression for the same women, may be driven by a need for comfort and affection; thus, sex may become a strategy to deal with negative feelings and emotions (as suggested by Bancroft et al., 2003b). In these two examples, there are different mechanisms used to explain the same outcome in sexual desire observed across different mood states: increased sexual desire as a consequence of celebration (e.g., a work promotion, an anniversary, or just to positively end the Saturday-night—see the “weekend effect” found in Roney & Simmons, 2013) vs. increased sexual desire as need for comfort (e.g., because you just had a fight with a friend, or a bad interview).

On the other hand, it is also possible that a single common mechanism can be used to explain similar changes in sexual desire across different extreme mood states. This idea is best captured by interactions/moderating effects. To incorporate this possibility, we propose that in addition to distinct mechanisms causing similar changes in sexual desire at the opposite extremes of mood (as discussed in the previous paragraph), in some cases there may be interactive effects involving negative mood and some moderator variable, thus providing a common mechanism for similar changes in sexual desire at the opposite extremes of mood. These potential moderating effects are the focus of the following paragraphs.

### Moderating Effects of Sensation Seeking

One plausible common mechanism responsible for increased (or decreased) sexual desire at both extremes of depressed mood is sensation seeking. There are three mechanisms by which women high on sensation seeking might experience increased sexual desire for both high and low levels of negative mood. First, women may mislabel general arousal as sexual arousal, and because sensation seekers actively pursue intense emotions to increase their arousal, and would thus live in a state of increased general arousal, they will have more opportunity to misinterpret high levels of general arousal as sexual arousal (and because women tend to not distinguish between sexual arousal and sexual desire—see Graham et al., 2004; Rosen et al., 2000, they would be more likely to experience feelings of sexual arousal also as sexual desire). This mechanism may apply across diverse mood states. Specifically, when high sensation seeking women experience high or low levels of depression, they may pursue intense emotional experiences to either alleviate the depression or enhance the emotional experience of their low depression state, thus increasing their level of arousal, which could then be perceived as increased sexual desire. Second, it is also possible that when pursuing intense emotions (at both extremes of negative mood), and thus increasing their level of general arousal, these women may also objectively be more prone to responding to sexual cues (because their average level of general arousal is typically high, they would require less stimulation to reach a threshold for responding to sexual cues) and thus to experience increases also in sexual arousal and desire. Third, high sensation seekers might have learned to associate sex with inducing intense emotions and high general arousal, such that sex becomes one way of pursuing experiences leading to intense emotions, both in case of improving negative mood and increasing positive mood. Therefore, it could be expected that the association between negative mood and sexual desire, across the full range of mood, would depend on the individual’s level of sensation seeking.

Previous research has examined this idea and has shown only weak associations between sensation seeking (primarily the disinhibition sub-scale) and the paradoxical effect of mood on sexuality (i.e., high scores on the *Mood and Sexuality Questionnaire;* or MSQ), for both heterosexual (Bancroft et al., 2003b) and gay men (Bancroft et al., 2003c). Yet, stronger associations have been found between sensation seeking (again, primarily the disinhibition sub-scale), and risky sexual activity, for both heterosexual (Bancroft et al., 2004), and gay men (Bancroft et al., 2003a). However, because the current study and interpretations are focused on women’s sexual desire rather than men’s, and because the paradoxical effect in the current study was examined at both ends of the mood continuum rather than only the high end of negative mood, results could be significantly different, and thus, future research should further test for these associations with women, and considering the full range of mood symptoms.

### Moderating Effects of Erotophobia–Erotophilia

An individual’s position along the personality dimension of erotophobia–erotophilia (Fisher et al., 1988) could also moderate the association between mood and sexual desire and thus provide a second common mechanism responsible for increased (or decreased) sexual desire at both extremes of negative mood. Erotophobia–erotophilia is a person’s propensity to respond to sexual cues with either positive (approach) or negative (avoidance) affective evaluations and is proposed to develop primarily based on socialization (Fisher et al., 1988). However, in addition to socialization, it is also likely that classical conditioning of associations between the individual’s emotional and behavioral response (e.g., "I had sex when I was really anxious”), and the positive or negative outcome of that experience (e.g. “I felt relaxed” or “I felt even more anxious”), also contribute to this propensity. It could therefore be hypothesized that strong emotions may be associated with—and thus triggers for—either seeking out sex (erotophilic tendency) or avoiding sex (erotophobic tendency), depending on past experiences of having sex in moments of high emotional states.

Note that, also based on learning and experience, and thus associated with an individual’s position on the erotophobia–erotophilia continuum, are diverse cognitive coping strategies that could be adopted to respond to extreme mood states. Therefore, even the subjective repertoire of sex-related coping strategies might play a role as a moderator in the association between mood and sexual desire.

## Final Conclusions

### Limitations

There are important limitations of this study that should be noted. Probably the most important limitation concerns the lack of data on type and quality of sexual relationships (e.g., casual sex, short-term, long-term, etc.) and whether participants were sexually active, all of which could be moderators of the observed paradoxical associations. Moreover, because participants could not be using hormonal contraceptives, the sample might be biased toward women without a partner and possibly less sexually active. An additional variable that may moderate the association between negative mood and sexual desire is whether the target of sexual desire is dyadic vs solitary sexual activity. The importance of distinguishing between solitary and dyadic sexual desire is underlined by research showing that depressed women, as compared to non-depressed women, report higher levels of desire for solitary sexual activity, but not for partnered sexual activity (see Frohlich & Meston, 2002).

Concerning aspects not directly related to sexuality, we did not specifically examine coping strategies that individuals use to manage negative mood, nor did we measure personality traits or differences in autonomic nervous system response. By directly assessing these variables, future research will hopefully be able to provide a better theoretical explanation of why women demonstrate such vastly different associations between negative mood and sexual desire.

Finally, the low rates of psychological diagnoses might suggest a biased sample, although we believe that it actually reflects an under-usage of mental health care in Italy. For example, while this study was conducted to examine menstrual cycle symptoms, none of the women indicated a diagnosis of PMS/PMDD, although examination of individual data suggests that some of them should have been. If these low rates are an artifact of mental health under use, then we simply lack full information on the mental health status of our sample. If this is true, then this lack of mental health information, beyond the current data collection, would be relevant because Cyranowski and colleagues (2004) found differences in sexual functioning between women with and without a history of depression, even after controlling for current depressive symptoms. This suggests that significant clinical depressive episodes should be considered in addition to daily reports. Moreover, although it is important to control for use of antidepressants given their potential effect on female sexual arousal (Lorenz & Meston, 2012), in the current study only one participant (< 1%) was taking antidepressants, and thus, the possibility that antidepressant use might have biased our results can be ruled out.

### Future research

The present study focuses on female university students. In order to further assess and understand the associations between sexual desire and negative mood, future research could focus on specific populations that so far have been overlooked in these kinds of studies. For example, Bancroft and colleagues (2003a) showed that a similar pattern of changes in sexual desire associated with increased and decreased negative mood was present in homosexual and heterosexual men, but there is still no study that has investigated this relationship specifically in lesbian women. In addition to individuals with different sexual orientation, further extension of this work should also focus on men and individuals outside of the cis-gender binary. Furthermore, it has been consistently found that sexual desire varies with age (Hällström & Samuelsson, 1990; Shifren, Monz, Russo, Segreti, & Johannes, 2008), and since the current study focuses on university students, it would be important to further investigate the relationship between negative mood and sexual desire among women across different ages. For example, future studies might consider studying the associations between mood and sexual desire in menopausal women, who report not only lower levels of sexual desire (Häillström & Samuelsson, 1990) but also higher levels of depressive and anxiety symptoms (Jafari, Hadizadeh, Zabihi & Ganji, 2014; Unsal, Tozun & Ayranci, 2011).

### Strengths

These limitations acknowledged, the current study also provides important strengths. Specifically, the use of daily reports of both mood symptoms and sexual desire for two full menstrual cycles (approximately two full months), controlling for menstrual cycle-related changes in sexual desire, and using within-person random effects modeling, are all important methodological advancements in this area specifically, and in sexology more broadly. Indeed, we believe that the methods and analyses used in this study are unprecedented in this area, and given the high methodological quality of this study, the results provide strong empirical support for the observed findings. We further suggest that one important strength of this study is the novel and compelling results that provide insights and new questions for future research.

### Final Comments

The present study has extended past research on the paradoxical associations between negative mood and sexual desire considering the full range of negative mood symptoms and using daily reports of all measures over two months of assessment. Important novel findings were that some women exhibit within-person paradoxical associations between negative mood and sexual desire across the full range of mood states, meaning they showed the same increase or decrease in sexual desire at both the high and low extremes of mood symptoms. Our interpretation of these results suggests that causal mechanisms of sexual desire are very idiosyncratic, requiring in-depth assessment of multiple factors, such as causes of mood symptoms, personality, past learning, coping strategies, and types of sexual or romantic relationships, that may moderate the effects of mood on sexual desire. Although testing for possible moderators will be methodologically demanding, we believe that to better understand these paradoxical associations, and sexual desire more broadly, it is both possible and necessary. Indeed, discussing average effects of mood on sexual desire, while overlooking individual differences, might limit, rather than expand, our understanding of human sexuality. We therefore suggest that individual differences in sexual response and sexual functioning should be the focus of future research—not just when discussing single cases inside the clinical settings, but also in behavioral and cognitive research, to then provide better diagnosis and more accurate indications for both research and therapy.

## Data Availability

Data are available upon request to the second author.
